# Bidimensional Increment Entropy for Texture Analysis: Theoretical Validation and Application to Colon Cancer Images

**DOI:** 10.3390/e27010080

**Published:** 2025-01-17

**Authors:** Muqaddas Abid, Muhammad Suzuri Hitam, Rozniza Ali, Hamed Azami, Anne Humeau-Heurtier

**Affiliations:** 1 Faculty of Computer Science and Mathematics, Universiti Malaysia Terengganu, Kuala Terengganu 21030, Malaysia; muqaddasabid3@gmail.com (M.A.); rozniza@umt.edu.my (R.A.); 2 Centre for Addiction and Mental Health, University of Toronto, Toronto, ON M6J 1H1, Canada; hmdazami@gmail.com; 3 LARIS, SFR MATHSTIC, Univ Angers, F-49000 Angers, France; anne.humeau@univ-angers.fr

**Keywords:** biomedical imaging, multiscale increment entropy, texture analysis, texture irregularity, two-dimensional increment entropy

## Abstract

Entropy algorithms are widely applied in signal analysis to quantify the irregularity of data. In the realm of two-dimensional data, their two-dimensional forms play a crucial role in analyzing images. Previous works have demonstrated the effectiveness of one-dimensional increment entropy in detecting abrupt changes in signals. Leveraging these advantages, we introduce a novel concept, two-dimensional increment entropy (IncrEn2D), tailored for analyzing image textures. In our proposed method, increments are translated into two-letter words, encoding both the size (magnitude) and direction (sign) of the increments calculated from an image. We validate the effectiveness of this new entropy measure by applying it to MIX_2*D*_(*p*) processes and synthetic textures. Experimental validation spans diverse datasets, including the Kylberg dataset for real textures and medical images featuring colon cancer characteristics. To further validate our results, we employ a support vector machine model, utilizing multiscale entropy values as feature inputs. A comparative analysis with well-known bidimensional sample entropy (SampEn_2*D*_) and bidimensional dispersion entropy (DispEn_2*D*_) reveals that IncrEn_2*D*_ achieves an average classification accuracy surpassing that of other methods. In summary, IncrEn_2*D*_ emerges as an innovative and potent tool for image analysis and texture characterization, offering superior performance compared to existing bidimensional entropy measures.

## 1. Introduction

Entropy, as defined in information theory by Shannon in 1949, quantifies the level of uncertainty present in data [1]. It measures the amount of information contained within a variable. Extracting information from a variable is of significant interest to scientists across various fields (see, e.g., [2,3,4]). In the context of information theory, several entropy measures, e.g., approximate entropy (ApEn) [5], sample entropy (SampEn) [6], permutation entropy (PermEn) [7], distribution entropy (DistrEn) [8], dispersion entropy (DispEn) [9], and increment entropy (IncrEn) [10] have been developed to extract meaningful information from unidimensional signals. These entropy measures allow scientists to analyze and understand the underlying patterns, complexity, and dynamics present in the data [11]. However, new one-dimensional entropy measures are still being proposed to overcome the drawbacks of existing ones.

ApEn [5] assesses pattern regularity by measuring the likelihood that similar patterns of a certain length will remain similar when compared to other subsequences. However, ApEn has some drawbacks. It tends to exhibit a bias toward regularity because of self-matching, lacks relative consistency, and is sensitive to data length. SampEn [6] addresses these issues by excluding self-matches and demonstrates relative consistency. It is less dependent on data length. However, for very short data, SampEn may still lead to unreliable or undefined values. PermEn was also developed as a complexity measure based on ordinal data to quantify the complexity of the given data [7]. Following that, Liu and Wang [12] proposed fine-graining PermEn. By fine-graining the partition, this modified version of PermEn responds more quickly to abrupt changes in amplitude. However, PermEn only considers the order of values in a time series, disregarding changes in magnitude between the elements. Even in the modified version of PermEn, when the data have equal-value elements, it either ignores them or treats them all as one symbol. Besides this, other entropy measures for complexity analysis were introduced like distribution entropy [8] and dispersion entropy [9], but none of them take into account the changes in magnitude between adjacent elements.

To address these limitations, IncrEn was proposed by Liu et al. [10]. IncrEn ranks the magnitudes of the variations between adjacent elements based on a precision factor and the standard deviation of the data. This quantifies the changes in magnitude between adjacent elements and also considers the order of the elements. IncrEn captures the local dynamics and changes between consecutive data points, making it effective for detecting abrupt changes or irregular patterns in the data. Compared to SampEn and PermEn, IncrEn is more adept at capturing and characterizing the structural information present in time series data [13]. IncrEn addresses the issue of undefined values that can occur in SampEn calculations. IncrEn does not suffer from the equal-value problem encountered in some other entropy measures. Moreover, it demonstrates robustness to noise, making it more suitable for analyzing noisy or complex data. Liu et al. [10] compared IncrEn with SampEn and PermEn, investigating the impact of Gaussian noise. They found that IncrEn displayed greater sensitivity to hidden changes in time series compared to PermEn, which treated analogous patterns as identical. Furthermore, both IncrEn and PermEn exhibited good invariance when applied to Gaussian noise sequences, whereas the SampEn results fluctuated significantly [10]. Furthermore, IncrEn showed better performance in seizure detection from real epileptic EEG signals [10]. It also performed well in detecting fault vibration signals [10].

Entropy measures for graph signals have recently been designed [14,15]. Some two-dimensional (2D) entropy algorithms have also been proposed recently to estimate the irregularity of textures or images (see, e.g., [16,17,18,19,20]). Three-dimensional measures have been published too [21]. The two-dimensional entropy measures have been applied to solve many texture problems in multiple domains [22]. Although this research area is still recent, the results obtained with the bidimensional entropy measures are very promising in various domains (see, e.g., [23,24]). The 2D measures can be used to study texture and analyze the irregular structures of images in a similar way to that implemented for signals [25]. Until now, few of the bidimensional entropy measures have been implemented, and many tasks are still to be explored in this way. Moreover, multiscale entropy measures have also emerged as a significant tool in the domain of image analysis, providing a sophisticated means to assess intricate patterns within images. These measures allow us to explore the complex nature of images by quantifying their information across multiple scales.

Recently, multivariate multiscale increment entropy (MMIE) was introduced by Wang et al. [13]. In [26], they proposed the multiscale IncrEn (MIE) and showed that MIE aligns better with the complexity loss theory of disease and aging in various physiological signals at different scales compared to the popular multiscale SampEn (MSE) method and refined composite multiscale dispersion entropy (RCMDE). MIE exhibits superior discrimination ability for physiological conditions affecting time series complexity, and its robustness to parameters outperforms MSE and RCMDE. Additionally, MIE is the most efficient in terms of time consumption, followed by RCMDE and MSE [26].

Due to the above-mentioned advantages of IncrEn in comparison with PermEn or SampEn, we introduce two-dimensional increment entropy (IncrEn_2*D*_). Inspired by the ability of IncrEn in detecting changes in both the size (magnitude) and direction (sign) of the increments calculated from a signal, we believe that this extension, along with its multiscale variant for images, holds promise for texture pattern analysis. This approach may offer advantages such as better characterization of structural information, improved discrimination ability for diverse physiological conditions, and enhanced robustness to noise and parameter variations.

This new measure was first studied through the analysis of synthetic images. Then, real datasets, including colon cancer images, were processed, and the results were compared with those of other, already existing 2D entropy-based measures.

## 2. Methods

In the following, the method we propose (IncrEn_2*D*_) is described, together with its multiscale version. The datasets processed and the computational steps are also detailed.

### 2.1. Two-Dimensional Increment Entropy, IncrEn_2D_

The new entropy measure that we propose here, IncrEn_2*D*_, is the extension of 1D IncrEn [10] to its bidimensional form. Its algorithm is defined as follows. Let us consider a grayscale image **I** represented as a matrix of size W×H, where *W* is the width (rows) and *H*, the height (columns). The calculation of IncrEn_2*D*_ for the given image **I** consists of the following steps:

1. First, an increment image **V**(**I**) is formed from the original image **I**. This can be performed in two different ways, as illustrated in Figure 1:
  (a) Row-wise increment image. In this case, pixel values are subtracted in adjacent rows so the size of the increment image would be (W−1)×H.
  (b) Column-wise increment image. In this case, pixel values are subtracted in adjacent columns, and thus, the increment image would be W×(H−1).

**Figure 1:**
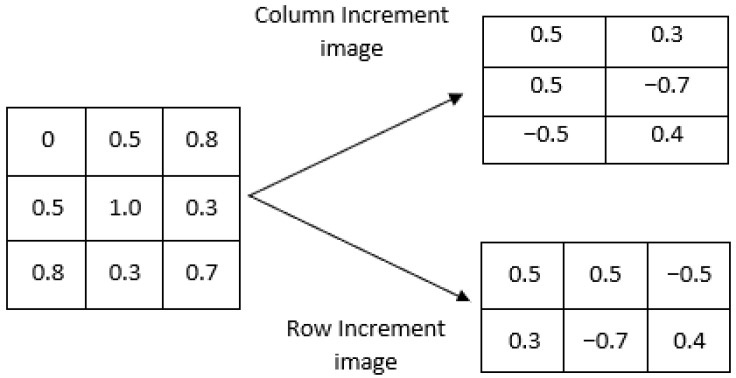
Illustration of the two possible methods for constructing the increment image **V**(**I**).
2. Given a positive integer *m*, divide the increment image into overlapping blocks of size m×m (see Figure 2, where m=2). We define **V**_m_(*i*,*j*) as the *m*-length square block in image **V**(**I**), where the indices range from row *i* to i+m−1 and from column *j* to j+m−1. The total number of blocks **$N_{m}$** can be computed as ((W−1)−m+1)×(H−m+1) for a row-wise increment image and (W−m+1)×((H−1)−m+1) for a column-wise increment image.
3. Vectorize each block to obtain an increment vector **V**_m_(*i*,*j*) and calculate the *n*th unique word sequence **W**_*n*_ for each **V**_m_(*i*,*j*), for a given quantifying resolution *R*. Each element of the increment vector **V**_m_(*i*,*j*) is mapped into word pattern composed of two parts: sign $s_{m}\left(i,j\right)$ and magnitude $q_{m}\left(i,j\right)$. The sign function produces different results depending on the input element. If the input element is greater than 0, the function returns 1. If the input element is equal to 0, the function returns 0. If the input element is less than 0, the function returns −1. The magnitude that represents the extent of difference between these neighboring pixels is determined by the resolution parameter *R*. Subsequently, every increment vector is transformed into a pattern vector comprising $2\times m^{2}$ elements (considering both sign and magnitude). To gain a complete understanding of creating a word pattern, let us consider the example of the template vectors illustrated in Figure 3, where the quantifying resolution *R* is taken as 4. The sign is calculated as $s_{m}\left(i,j\right)$ = sign(**V**_m_(*i*,*j*)), and magnitude $q_{m}\left(i,j\right)$ is calculated using the following equation:
  (1) $$q_{m}\left(i,j\right)=\left\{\begin{array}{cc} 0 & \;\mathrm{if}\;\mathrm{std}(V_{m}\left(i,j\right))=0\\ min(R,\left\lfloor \frac{V_{m}\left(i,j\right)\times R}{\mathrm{std}(V_{m}\left(i,j\right))}\right\rfloor ) & \;\mathrm{if}\;\mathrm{std}(V_{m}\left(i,j\right))\neq 0\; \end{array}\right.$$
4. Count the total number of instances $Q(W_{n})$ of every unique *n*th word pattern $W_{n}$.
5. Compute the relative frequency of each unique word using the following equation:
  (2) $$F\left(W_{n}\right)=\frac{Q(W_{n})}{N_{m}},$$
  where $N_{m}$ is the total number of words for the given increment image.
6. Calculate the two-dimensional increment entropy, IncrEn_2*D*_, of image **I** using the following equation:
  (3) $${\mathrm{IncrEn}}_{2D}\left(I,m,R\right)=-\sum_{n=1}^{{\left(2R+1\right)}^{m^{2}}}F\left(W_{n}\right)\log F\left(W_{n}\right).$$

With a specific *m* and *R*, each increment vector yields ${\left(2R+1\right)}^{m^{2}}$ distinct patterns. Thus, IncrEn_2*D*_ is bounded within $\left[0,m^{2}\times \log\left(2R+1\right)\right]$. While this upper bound of IncrEn_2*D*_ reflects theoretical maximum information content, it is important to note that the formulation of IncrEn_2*D*_ diverges from classical entropy principles. While classical Shannon entropy focuses on individual pixels, IncrEn_2*D*_ extends this concept by analyzing blocks of pixels and is influenced by a quantification parameter (*R*). Thus, while it retains elements of Shannon entropy, it introduces a novel approach that considers spatial dependencies within images.

### 2.2. Multiscale 2D Increment Entropy

One- and two-dimensional entropy measures are commonly employed to quantify the irregularity within signals or images at a specific scale. These methods are adept at identifying repetitive structures and are particularly effective in scenarios of complete randomness, such as white noise. However, their sensitivity to high-frequency components can lead to an oversight of multiple inherent scales in the data [17]. To address this limitation, researchers have introduced multiscale entropy-based approaches. These methods have demonstrated interesting results in the domain of texture classification (see, e.g., [16]). Moreover, they offer the capability to evaluate irregularities across different scales, providing a comprehensive insight into the complexity of a given signal or image. Therefore, we herein propose the multiscale IncrEn_2*D*_ (MIncrEn_2*D*_) to measure the complexity of image textures at different scale factors τ. The two-step process for calculating MIncrEn_2*D*_ is described as follows:

1. Coarse-graining procedure: The non-overlapping moving-average coarse-graining procedure is used in this study to divide the image into multiple scales. In this procedure, a non-overlapping window of size τ sweeps across the entire image, and the pixels within each window are averaged. These resulting average values shape the coarse-grained images. These images, while not being the subsets of the original, encapsulate information about the entire original image. When considering an image **I** of dimensions W×H, a coarse-grained image **G**^(τ)^ at scale factor τ is expressed mathematically as follows:
  (4) $$G_{i,j}^{\left(\tau \right)}=\frac{1}{\tau^{2}}\sum_{k=(i-1)\tau +1,l=(j-1)\tau +1}^{k=i\tau ,l=j\tau }I_{k,l},$$
  where $1\leq i\leq \left\lfloor \frac{H}{\tau }\right\rfloor ,1\leq j\leq \left\lfloor \frac{W}{\tau }\right\rfloor$, and τ is the scale factor. For scale factor 1, the coarse-grained image corresponds to the original image. At higher scale factor, the dimensions of the coarse-grained image are reduced by a factor of τ.
2. Application of IncrEn_2*D*_: In the subsequent step, IncrEn_2*D*_ is applied individually on each of the coarse-grained images.

### 2.3. Evaluation Images and Computational Steps

To evaluate the performances of IncrEn_2*D*_, MIX_2*D*_(*p*) processes, as well as artificial periodic and synthesized textures, were used. Real datasets were also processed and studied.

#### 2.3.1. Two-Dimensional MIX Process (MIX_2*D*_(*p*))

**Figure 4:**
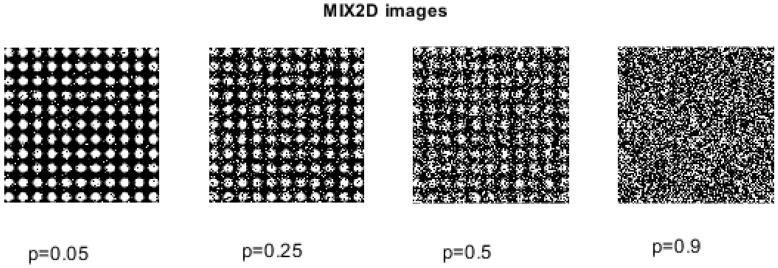
MIX_2*D*_(*p*) family of images with varying noise levels, p.

The creation of MIX_2*D*_(*p*) processes is based on the definition of its unidimensional version, MIX_1*D*_(*p*). MIX_2*D*_(*p*) denotes images where the parameter *p* corresponds to the level of white noise. As *p* goes from 0 (representing completely periodic sinusoidal images) to 1 (indicating highly irregular images), the images of size W×H exhibit different degrees of spatial irregularity, described as ${\mathrm{MIX}}_{2D}{\left(p\right)}_{i,j}=\left(1-z_{i,j}\right)x_{i,j}+z_{i,j}y_{i,j}$, i=1,2,…,H, j=1,2,…W, where $x_{i,j}=\sin\left(\frac{2\pi i}{12}\right)+\sin\left(\frac{2\pi j}{12}\right)$, and $y_{i,j}$ represents a uniform two-dimensional matrix of white noise, spanning from $-\sqrt{3}$ to $\sqrt{3}$. In this context, $z_{i,j}$ represents a random variable that takes the value 1 with a probability of *p* and the value 0 with a probability of 1−p [25]. Consequently, as the value of *p* increases, the images become more irregular; see Figure 4.

To assess the effectiveness of IncrEn_2*D*_ in quantifying images with varying degrees of irregularity and randomness, different MIX_2*D*_(*p*) images were created and used.

#### 2.3.2. Artificial Periodic and Synthesized Texture Images

To assess the behavior of IncrEn_2*D*_ during the transformation of periodic textures into their synthesized counterparts, four sets of periodic textures and their corresponding synthesized textures were used from [27].

#### 2.3.3. Image with Additive Noise

To examine the influence of two-dimensional white Gaussian noise (WGN_2*D*_) and salt-and-pepper noise (SPN_2*D*_) on IncrEn_2*D*_, we conducted an evaluation using the widely recognized Lena image with size 256 × 256 pixels, as a standard reference. To achieve image normalization, we applied a two-step process. After converting the image to its grayscale form, we subtracted the mean of the image from each pixel value. Next, we divided the resulting values by the standard deviation of the image. Following the normalization of the image within the range of 0 to 1, we systematically introduced various levels of WGN_2*D*_ with a mean equal to 0 and variance values of 0.01, 0.03, 0.05, and 0.07. The noise was added to almost every pixel of the image. Furthermore, we integrated SPN_2*D*_ with noise density values of 0.01, 0.05, and 0.09 into the aforementioned normalized reference image. In the case of SPN_2*D*_, the noise density, *d*, determines the amount of noise applied to the image, with *d* being multiplied by the number of pixels.

#### 2.3.4. Effect of Different Standard Deviation Computations on IncrEn_2*D*_

We also explored the influence of the standard deviation computation in Equation (1) on the IncrEn_2*D*_ results using MIX_2*D*_(*p*) processes. More precisely, we studied IncrEn_2*D*_ when the standard deviation of the increment block (**V**_m_(*i*,*j*)) is used and when the standard deviation of the whole increment image V(I) is used in Equation (1).

#### 2.3.5. Effect of Row-Wise and Column-Wise Increment Images

**Figure 5:**
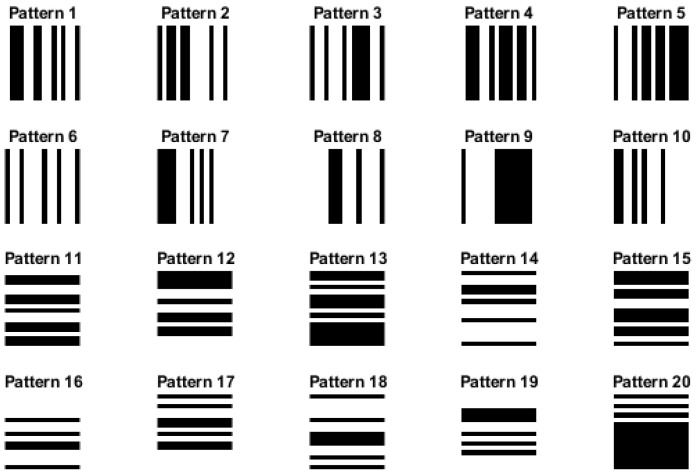
Simulated images of size 256 × 256 pixels with vertical stripes (patterns 1–10) and horizontal stripes (patterns 11–20).

As mentioned above, the two main approaches used to calculate the increment images (the first step of the algorithm) are the row-wise and column-wise increment methods. In this work, we evaluated the influence of these two different ways of computation by creating simulated images with vertical and horizontal stripes (see Figure 5). For the vertical and horizontal stripe pattern images, we set the image dimensions to 256 × 256 pixels, set the stripe width (vertical stripes) and strip height (horizontal stripes), and calculated the number of stripes based on the image width. Then, we generated ten different patterns of each category by creating random binary pattern arrays, where each element represents the color of a stripe (0 for black, 1 for white). We constructed the vertical and horizontal stripe pattern images using these patterns by setting pixel values accordingly. We also carried out an experiment with pure white and black images, where all pixel values were equal to 255 for pure white and 0 for pure black, to evaluate the performance of our proposed method in these extreme cases.

#### 2.3.6. Kylberg Real Texture Dataset

**Figure 6:**
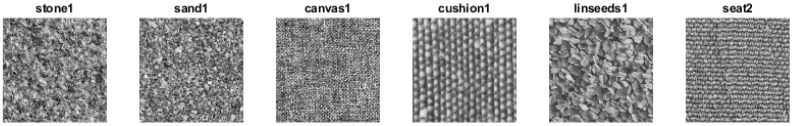
One image (576 × 576 pixels) of each of the six selected categories from the Kylberg dataset.

In this study, a portion of the Kylberg texture dataset was used. The dataset can be accessed publicly from [28]. Specifically, we chose six distinct categories of images. These images represent various fabrics and surfaces: canvas1, cushion1, linsseeds1, sand1, stone1, and seat2 (see Figure 6. Each sample is 576 × 576 pixels in size).

#### 2.3.7. Colon Cancer Dataset

**Figure 7:**
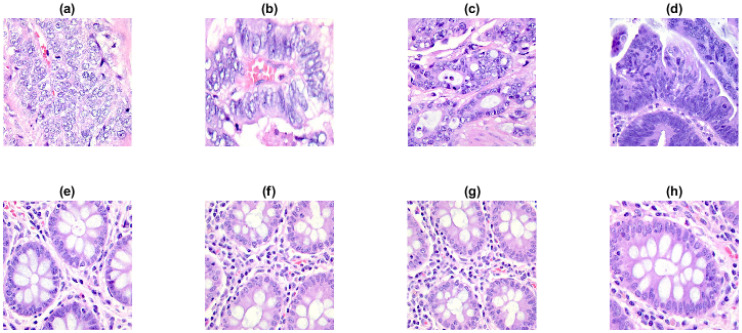
A few sample images (768 × 768 pixels) from the LC25000 dataset: (**a**–**d**) colon cancer tissues and (**e**–**h**) normal tissues.

Colon adenocarcinoma stands out as the most prevalent forms of colon cancer, accounting for over 95% of all colon cancer instances. It occurs when a specific type of tissue growth, known as adenoma, develops in the large intestine, eventually transforming into cancer [29]. Multiscale increment entropy (MIncrEn_2*D*_) was applied on part of the lung and colon cancer histopathological image dataset known as the LC25000 dataset for the validation of the approach. This dataset comprises 25,000 color images showcasing five distinct lung and colon tissue types [30]. For this study, we only considered a portion of the colon cancer part (160 images of cancerous and normal tissue). Some samples of the cancerous and normal tissues from the dataset are presented in Figure 7.

The original images, initially sized at 768×768 pixels, were preprocessed to ensure uniformity and reduce computational load prior to entropy calculation. During this stage, all images were converted to grayscale images and resized to 128×128 pixels using bilinear interpolation prior to entropy calculation. Multiscale analysis considered scale factors of up to 4, resulting in the smallest image size of 32×32 pixels. MIncrEn_2*D*_ provided entropy values across all scales for both cancerous and normal tissues. This study utilized these entropy values as features for classifying tissues, employing a support vector machine (SVM) with a radial basis kernel function due to its ability to capture intricate relationships. The dataset comprised 160 histopathological images from LC25000, evenly divided between cancerous and normal tissues. Out of these, 70% of the dataset (112 images) were allocated for training, while the remaining 30% (48 images) were reserved for testing. The primary features for classification were entropy values, derived from 4 different scale factors. The SVM classifier was trained on the training dataset and evaluated using the test dataset. This procedure was repeated 5 times and took the average values to ensure consistency in evaluation. To evaluate the classifiers performance, metrics such as the classification accuracy, precision, recall, and F1-score were computed. The results obtained using features given by IncrEn_2*D*_ were compared with the results given by the now well-known multiscale bidimensional sample entropy (SampEn_2*D*_) [25] and multiscale bidimensional dispersion entropy (DispEn_2*D*_) [19] measures.

## 3. Results

This section details the results obtained from the aforementioned images.

### 3.1. Two-Dimensional MIX Process (MIX_2D_(p))

**Figure 8:**
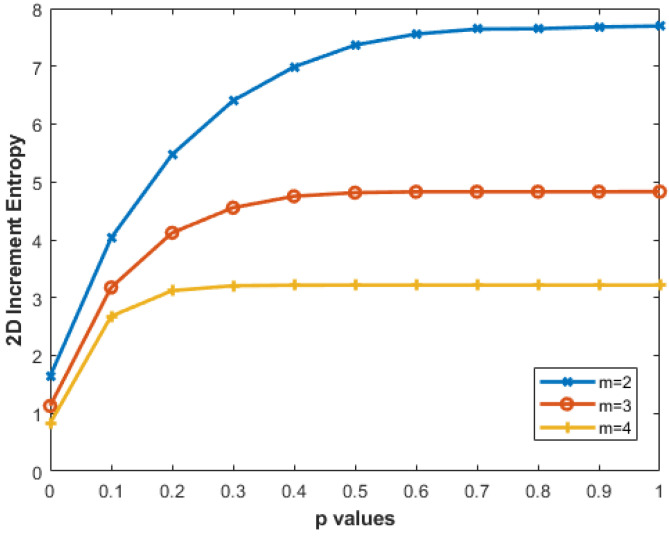
IncrEn_2*D*_ for MIX_2*D*_(*p*) images of size 256 × 256 pixels for *R*=4 and m=2,3, and 4.

We created 256 × 256 pixel MIX_2*D*_(*p*) images by varying the parameters—m=2,3,4 and *p* = 0 to 1 with a step size of 0.05—resulting in 21 different *p* values. Figure 8 illustrates that higher *p* values correspond to greater IncrEn_2*D*_ entropy. The entropy increase from p=0 to p=1 decreases with higher *m*: for m=2, the range is 1.6435 to 7.6724, while for m=4, it is 0.8280 to 3.2162. Smaller block sizes (e.g., m=2) capture finer details, while larger sizes encompass broader patterns.

**Figure 9:**
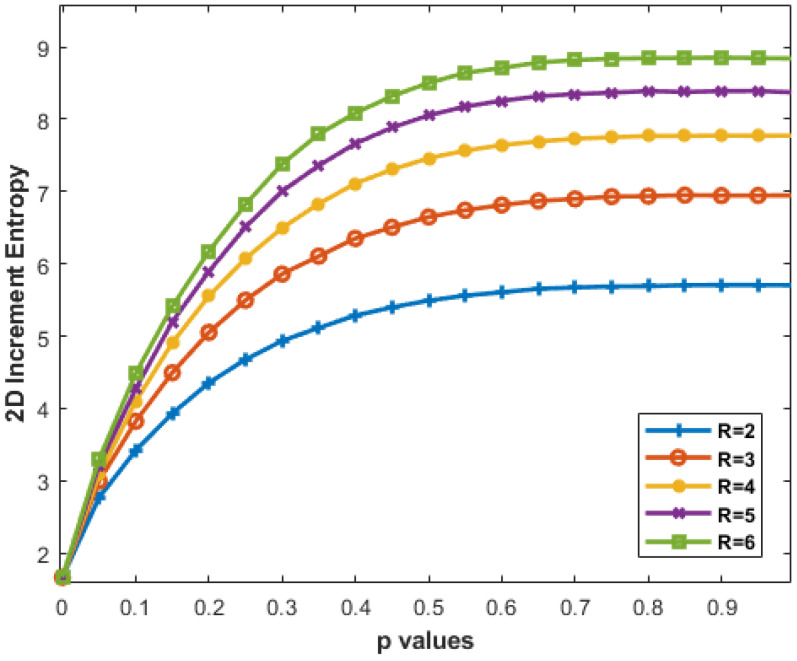
IncrEn_2*D*_ for MIX_2*D*_(*p*) images of size 256 × 256 pixels for m=2 and varying *R* values from 2 to 6.

Figure 9 shows that increasing *R* from 2 to 6 results in a steeper curve, especially notable for R=4,5,6. Higher *R* values provide finer detail, but diminishing differences suggest a saturation point where increased resolution has limited impact on pattern understanding.

**Figure 10:**
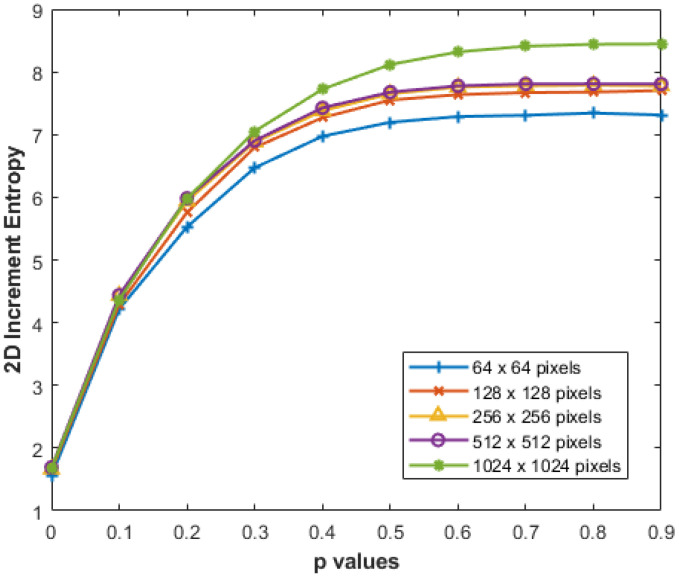
IncrEn_2*D*_ for MIX_2*D*_(*p*) images of different sizes for m=2 and R=4.

We conducted additional experiments to assess the reliability of our proposed IncrEn_2*D*_ across varying image sizes, aiming to verify its performance in measuring image irregularity. Specifically, we computed IncrEn_2*D*_ for MIX_2*D*_(*p*) while progressively increasing image sizes from 64×64 to 1024×1024 pixels, with m=2, R=4, and *p* varying from 0 to 1. The results, as depicted in Figure 10, consistently demonstrate stable results with changes in image size.

### 3.2. Artificial Periodic and Synthesized Texture Images

The results obtained with the artificial periodic and synthesized textures are presented in Table 1. We observe that IncrEn_2*D*_ values are greater for the synthesized textures compared to their corresponding periodic textures.

### 3.3. Image with Additive Noise

Table 2 presents the IncrEn_2*D*_ values obtained from the Lena image at different levels of WGN_2*D*_ and SPN_2*D*_. The results indicate that when higher variance values are applied to WGN_2*D*_, IncrEn_2*D*_ values also increase. Similarly, a higher noise density in SPN_2*D*_ leads to higher entropy values.

### 3.4. Effect of Different Standard Deviation Computations on IncrEn_2D_

**Figure 11:**
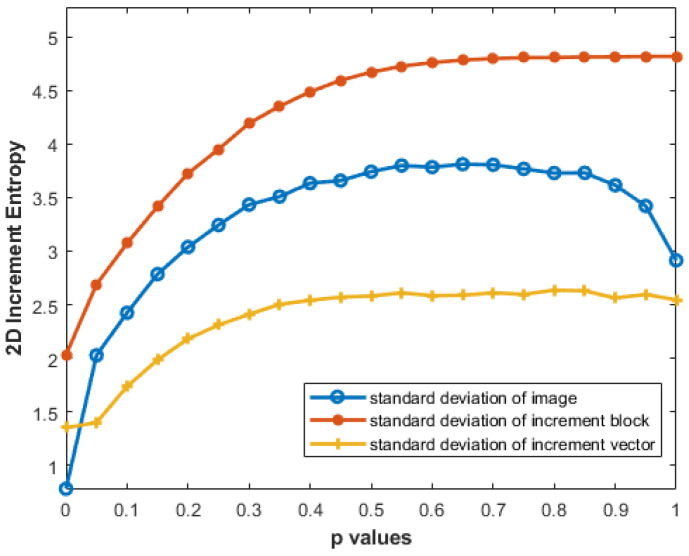
Effect of different standard deviations on IncrEn_2*D*_ values with m=2 and R=4.

It is evident from Figure 11 that the results obtained with the standard deviation of the original image **I** are somewhat misleading: the entropy values for MIX_2*D*_(0.9) and MIX_2*D*_(1) are lower than those of MIX_2*D*_(0.8). The standard deviation of increment block (**V**_m_(*i*,*j*)) provides better entropy estimates than the standard deviation of increment image (**V**(**I**)). The discrepancy arises from how the standard deviation is computed: the whole increment image considers all pixels, yielding a larger variance, while the increment block focuses on a fraction, providing a more localized measure of variation.

Figure 11 presents the effect of different standard deviation computations on IncrEn_2*D*_.

### 3.5. Effect of Row-Wise and Column-Wise Increment Images

**Figure 12:**
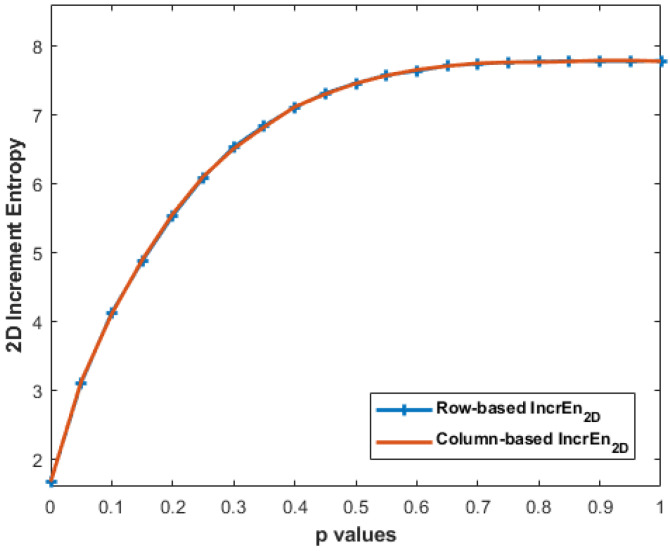
IncrEn_2*D*_ values with row-wise and column-wise increment images for MIX_2*D*_(*p*) of size 256 × 256 pixels with m=2 and R=4.

Despite the differences in the calculation, it can be observed, through Figure 12, that the IncrEn_2*D*_ values obtained with MIX_2*D*_(*p*) from both the row-wise and column-wise approaches are almost the same. This similarity in IncrEn_2*D*_ values can be attributed to the inherent characteristics of MIX_2*D*_(*p*) images. The results obtained with horizontal and vertical stripe pattern images are presented in Table 3.

As anticipated, the experiment with pure white and black images resulted in IncrEn_2*D*_ values of 0, as these types of images exhibit no irregularities.

### 3.6. Kylberg Real Texture Dataset

Table 4 presents the IncrEn_2*D*_, SampEn_2*D*_, and DispEn_2*D*_ values for the selected Kylberg texture groups. The results indicate distinct entropy values for the six selected groups with almost all entropy measures except that SampEn_2*D*_ shows minor differences between cushion1 and linseeds1. The same is observed for DispEn_2*D*_ with sand1 and stone1.

### 3.7. Colon Cancer Dataset

**Figure 13:**
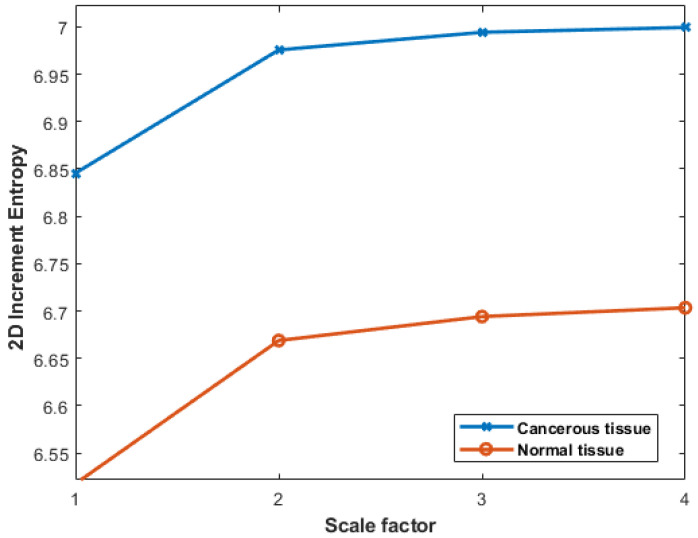
Multiscale IncrEn_2*D*_ values with m=2, R=4, and scale factors from 1 to 4 for cancerous and normal tissues.

The anatomical and biological characteristics of colon cancer often exhibit distinct horizontal arrangements along the colon length. For this reason, in the present experimentation, the preference was to employ row-wise increment images. The results obtained with colon cancer data can be seen in Figure 13. We observe lower IncrEn_2*D*_ values for normal colon tissues and higher IncrEn_2*D*_ values for cancerous tissues, irrespective of the scale factor used. This relationship between IncrEn_2*D*_ and tissue types implies its potential as a valuable tool in distinguishing between normal and cancerous colon tissues.

Moreover, the results based on the SVM model show an average classification accuracy of 77.22%, compared to SampEn_2*D*_ and DispEn_2*D*_, which achieved average classification accuracies of 59.31% and 47.63%, respectively, obtained using the same model (see Table 5).

### 3.8. Computational Time

In this study, we also conducted a comparative analysis for IncrEn_2*D*_, SampEn_2*D*_, and DispEn_2*D*_ in terms of computational time across different image sizes ranging from 50×50 to 200×200 pixels. We used MATLAB R2024a on a personal computer equipped with an Intel(R) Core(TM) i7-5600U CPU operating at 2.60 GHz and 8 GB RAM. To achieve this, we utilized images of varying dimensions from the Kylberg dataset. The results are presented in Table 6. We notice that DispEn_2*D*_ exhibits the shortest computational time, even with larger image sizes. IncrEn_2*D*_ closely follows, maintaining its computational advantage for small- to moderate-sized images. SampEn_2*D*_ incurs the highest computational time, particularly with larger image sizes.

## 4. Discussion

The results of this work demonstrate that IncrEn_2*D*_ offers superior performance in texture analysis across various image sizes and noise conditions, providing insights into its effectiveness for diverse image types and patterns. With the MIX_2*D*_(*p*) images, the behavior of IncrEn_2*D*_ with respect to the parameter *p* highlights its sensitivity to entropy changes. Specifically, higher *p* values correspond to greater IncrEn_2*D*_ entropy, suggesting that this parameter plays a key role in capturing the complexity of textures. Moreover, the observation that entropy increases from p=0 to p=1 decrease with higher block sizes *m*, illustrating the scale-dependent nature of this measure. Smaller block sizes, such as m=2, are more adept at capturing finer details, whereas larger block sizes, such as m=4, are better suited for recognizing broader patterns, aligning with previous findings in multiscale entropy analysis.

The impact of the threshold *R* is equally important, with higher values yielding finer detail, though the diminishing returns beyond R=4 suggest a saturation point. This result is crucial for practical applications, as it implies that increasing *R* beyond certain values may offer little additional benefit in terms of pattern recognition, potentially optimizing the computational cost for future implementations. Therefore, for the final selection of the *R* value, one can consider the trade-off between enhanced resolution and computational complexity. Based on the observed results, we can conclude that the optimal parameter set for further experimentation would be m=2 and R=4. This combination enables the IncrEn_2*D*_ to effectively quantify the irregularity of the images under analysis.

Our findings with synthetic and periodic textures reinforce the robustness of IncrEn_2*D*_, with synthesized textures consistently exhibiting higher entropy values than their periodic counterparts. This shows the discriminative power of IncrEn_2*D*_ in distinguishing between periodic and synthesized textures, while also providing a measure of the periodicity within the images. In other words, it provides a reliable method for differentiating between images that possess inherent periodic characteristics and those that have been artificially created or modified. The behavior of IncrEn_2*D*_ under noise conditions further validates its stability and utility. As the variance of 2D white Gaussian noise (WGN_2*D*_) increases, so does the IncrEn_2*D*_ value, confirming its sensitivity to added randomness in the image. Similarly, salt-and-pepper noise (SPN) at higher densities results in increased entropy values, reflecting its ability to quantify the complexity introduced by these types of distortions. This characteristic can be advantageous in real-world applications where noise is an inevitable factor, such as in medical imaging.

An important aspect of our analysis is the performance of IncrEn_2*D*_ with different standard deviation measures. The results demonstrate that the standard deviation of the increment vector provides the best differentiation of textures, outperforming both the standard deviation of the image itself and the increment image. This localized approach captures fine-grained entropy changes more effectively. Additionally, the block-based analysis introduces spatial locality, with the standard deviation of the increment block reflecting variations within a specific neighborhood, offering more insight into image structure and patterns. In contrast, the standard deviation of the whole increment image considers variations across the entire image, potentially diluting detailed information. Furthermore, the standard deviation of the increment vector (yellow curve in Figure 11) shows a smoother curve, indicating less sensitivity to localized changes within the image. This could result in a less steep entropy plot, as the measure focuses on overall patterns rather than reacting strongly to localized features.

Our experiments with row-wise and column-wise increment images on MIX_2*D*_(*p*) show a similar curve (see Figure 12). MIX_2*D*_(*p*) images tend to exhibit certain statistical properties, such as local spatial correlation and smooth intensity transitions. These properties result in a relatively balanced distribution of pixel differences in both horizontal and vertical directions. While the row-wise and column-wise increment images may emphasize different aspects of the image structure, their overall impact on IncrEn_2*D*_ entropy is comparable due to the statistical properties of MIX_2*D*_(*p*) images. The balance between horizontal and vertical variations ensures that the distribution of pixel differences is similar in both cases, leading to similar IncEn_2*D*_ values.

The results obtained with horizontal and vertical stripe pattern images (see Figure 5) emphasize the critical significance of considering texture orientation when applying IncrEn_2*D*_; see Table 3. The row-wise increment image is able to capture changes within each row, exhibiting sensitivity to horizontal variations and effectively detecting features and patterns extending horizontally across the image. Consequently, the row-wise increment image yields non-zero IncrEn_2*D*_ values for horizontal pattern images, while registering zero values for vertical pattern images. In contrast, the column-wise increment image is able to capture changes within each column, demonstrating sensitivity to vertical variations and in detecting features and patterns that extend vertically. As a result, the column-wise increment image yields non-zero IncrEn_2*D*_ values for vertical pattern images and, conversely, reports zero values for horizontal pattern images.

It is important to note that the choice between row-wise and column-wise increment images may depend on the specific characteristics of the image data or the analysis goals. For instance, if the image dataset predominantly contains horizontally oriented features or patterns, the row-wise increment image might provide more relevant information. Conversely, the column-wise increment image may be more informative if the dataset consists of vertically oriented features.

The application of IncrEn_2*D*_ to the Kylberg dataset revealed distinct entropy values for most texture groups, providing a strong basis for its use in texture classification tasks. The results suggest that IncrEn_2*D*_, like SampEn_2*D*_ and DispEn_2*D*_, could serve as a valuable tool for distinguishing between different patterns of fabrics and surfaces. They also highlight the potential ability of the measures for discriminating measures in texture analysis.

Finally, the colon cancer dataset results underscore the superior performance of IncrEn_2*D*_ in medical image analysis. Achieving an average classification accuracy of 77.22% with the SVM model, IncrEn_2*D*_ significantly outperformed SampEn_2*D*_ (59.31%) and DispEn_2*D*_ (47.63%) under the same conditions. Additionally, IncrEn_2*D*_ achieves the highest precision and sensitivity, indicating that, when it predicts positive cases (colon cancer), it is more accurate compared to SampEn_2*D*_ and DispEn_2*D*_. Moreover, it is better at capturing actual positive cases. On the other hand, SampEn_2*D*_ exhibits the highest specificity, suggesting that it performs better at correctly identifying negative cases compared to IncrEn_2*D*_ and DispEn_2*D*_. However, the major problem of SampEn_2*D*_ is that it can lead to undefined values at high-scale factors. DispEn_2*D*_ and IncrEn_2*D*_ do not have this undefined value problem. Moreover, the F1-score indicates that IncrEn_2*D*_ achieves a better balance between precision and sensitivity.

From a computational perspective, DispEn_2*D*_ exhibited the shortest computational time across all image sizes, followed closely by IncrEn_2*D*_. However, IncrEn_2*D*_ maintains a favorable balance between computational efficiency and performance, particularly for small- to moderate-sized images. In contrast, SampEn_2*D*_ incurs significantly higher computational costs, especially with larger images, which may limit its practical applicability in real-time or large-scale applications.

Though IncrEn_2*D*_ demonstrates effectiveness across different datasets, it is imperative to acknowledge its inherent limitations. Notably, the performance of IncrEn_2*D*_ may be hindered when dealing with large images (like 1024×1024 pixels), where computational efficiency may become a constraint. Additionally, it is pertinent to mention that IncrEn_2*D*_ currently operates solely on grayscale images, indicating a limitation in its applicability to colored images. These considerations underscore the need for further research to address these constraints and improve the applicability of the proposed method.

## 5. Conclusions

In this paper, we introduced a novel bidimensional entropy measure, IncrEn_2*D*_, and its multiscale form, to, respectively, estimate the irregularity and complexity of an image. These measures take into account both the size (magnitude) and direction (sign) of the increments calculated from an image. We studied their capability as powerful techniques for image analysis. We thoroughly examined the influence of the two parameters, namely the embedding dimension *m* and the quantification parameter *R*. We validated the performance of IncrEn_2*D*_ using MIX_2*D*_(*p*) processes, as well as artificial and synthesized textures. We also investigated the impact of different standard deviations and increment image choices on the IncrEn_2*D*_ calculation. Furthermore, the choice between row-wise and column-wise increment images should align with the specific image dataset’s predominant orientation or analytical objectives.

We validated IncrEn_2*D*_ on two real texture datasets: Kylberg and a medical dataset with colon cancer images. For Kylberg, IncrEn_2*D*_ effectively distinguished fabric patterns with distinct entropy values in designated groups, showing potential comparable to DispEn_2*D*_ and faster computation than SampEn_2*D*_. In biomedical images, IncrEn_2*D*_ differentiated normal and cancerous colon tissues across scale factors, outperforming multiscale SampEn_2*D*_ and multiscale DispEn_2*D*_ in accuracy and precision. This highlights IncrEn_2*D*_ as a reliable texture descriptor with broad relevance in biomedical applications. Overall, the introduced IncrEn_2*D*_ emerges as an innovative and possible tool for image analysis and texture characterization.

## Figures and Tables

**Figure 2:**
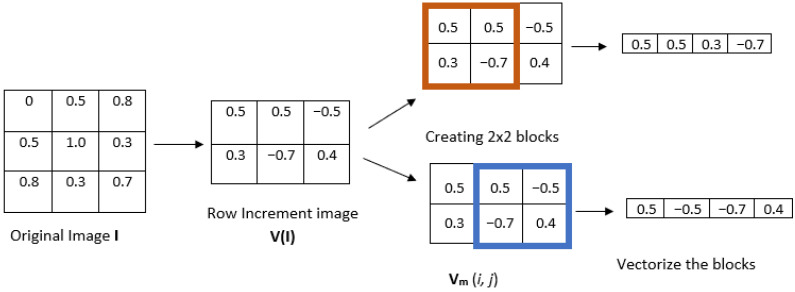
Illustration of steps 1 and 2 of IncrEn_2*D*_, using the row-wise method to compute the increment image.

**Figure 3:**
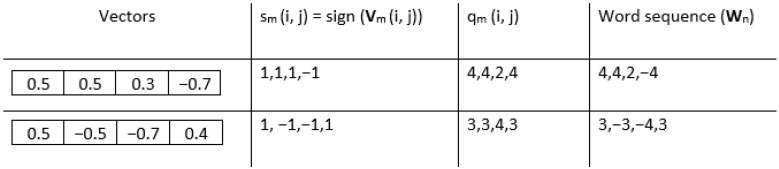
Template vectors illustrating the steps to obtain words.

**Table 1:** IncrEn_2*D*_ for artificial periodic textures and their synthesized textures.

Texture a	Texture b	Texture c	Texture d
3.6085	0.8242	3.2130	1.6720
Synthesized a	Synthesized b	Synthesized c	Synthesized d
3.8193	1.1930	4.0133	3.0262

**Table 2:** IncrEn_2*D*_ values for Lena image on which different levels of 2D white Gaussian noise (WGN_2*D*_) and salt-and-pepper noise (SPN_2*D*_) were added. Parameters were set as m=2 and R=4 using the column-wise increment image method and the standard deviation of a block for the IncrEn_2*D*_ calculation.

Type of Noise	Noise Level Addded	IncrEn_2*D*_
WGN_2*D*_	mean = 0, variance = 0.01	6.7972
WGN_2*D*_	mean = 0, variance = 0.03	6.8522
WGN_2*D*_	mean = 0, variance = 0.05	6.9075
WGN_2*D*_	mean = 0, variance = 0.07	6.9304
SPN_2*D*_	noise density = 0.01	2.3120
SPN_2*D*_	noise density = 0.05	2.6380
SPN_2*D*_	noise density = 0.09	2.9342

**Table 3:** IncrEn_2*D*_ values for vertical and horizontal stripes pattern images. Parameters were set as m=2 and R=4.

Vertical Stripe Images			Horizontal Stripe Images		
**Texture Image**	**Row-Wise Increment Image**	**Column-Wise Increment Image**	**Texture Image**	**Row-Wise Increment Image**	**Column-Wise Increment Image**
Pattern 1	0	0.3537	Pattern 1	0.2197	0
Pattern 2	0	0.3537	Pattern 2	0.2558	0
Pattern 3	0	0.1836	Pattern 3	0.3537	0
Pattern 4	0	0.1836	Pattern 4	0.1432	0
Pattern 5	0	0.2558	Pattern 5	0.3224	0
Pattern 6	0	0.3224	Pattern 6	0.3537	0
Pattern 7	0	0.3224	Pattern 7	0.2892	0
Pattern 8	0	0.2197	Pattern 8	0.3224	0
Pattern 9	0	0.2197	Pattern 9	0.2892	0
Pattern 10	0	0.3537	Pattern 10	0.2892	0

**Table 4:** IncrEn_2*D*_, SampEn_2*D*_, and DispEn_2*D*_ values for six different groups of texture surfaces from the Kylberg dataset. Parameters were set as follows: m=2 for all, R=4 for IncrEn_2*D*_, r=0.2 of the standard deviation of an image for SampEn_2*D*_, and c=3 for DispEn_2*D*_.

	Cushion1	Sand1	Linseeds1	Stone1	Canvas1	Seat2
IncrEn_2*D*_	5.0148	6.3386	6.4282	6.6797	7.6083	7.7559
SampEn_2*D*_	2.2899	4.3193	2.2025	4.9939	8.0463	6.1602
DispEn_2*D*_	2.4051	2.975	2.599	2.969	3.6843	3.1331

**Table 5:** Classification results obtained from the colon cancer data with the multiscale approaches (from scale factors 1 to 4) for IncrEn_2*D*_, SampEn_2*D*_, and DispEn_2*D*_ features.

	Multiscale	Multiscale	Multiscale
	IncrEn_2***D***_	SampEn_2***D***_	DispEn_2***D***_
Accuracy	77.22%	59.31%	47.62%
Precision	78.64%	62.68%	47.41%
Sensitivity	80.95%	24.20%	33.33%
Specificity	37.80%	62.20%	34.00%
F1-score	78.60%	61.03%	47.51%

**Table 6:** Comparison of computational time in seconds to calculate IncrEn_2*D*_, SampEn_2*D*_, and DispEn_2*D*_ on one image with different dimensions from the Kylberg dataset. Parameters were set as follows: m=2 for all, R=4 for IncrEn_2*D*_, r=0.2 × standard deviation of image for SampEn_2*D*_, and c=3 for DispEn_2*D*_.

Size (Pixels)	50×50	100×100	150×150	200×200
IncrEn_2*D*_	0.137	0.458	0.897	3.000
SampEn_2*D*_	0.562	2.733	15.760	58.914
DispEn_2*D*_	0.123	0.049	0.077	0.097

## Data Availability

The synthetic data generated and used in this study are available upon request. The real datasets analyzed in this study are publicly available.
